# A Comprehensive Survey of Indoor Localization Methods Based on Computer Vision

**DOI:** 10.3390/s20092641

**Published:** 2020-05-06

**Authors:** Anca Morar, Alin Moldoveanu, Irina Mocanu, Florica Moldoveanu, Ion Emilian Radoi, Victor Asavei, Alexandru Gradinaru, Alex Butean

**Affiliations:** 1 Faculty of Automatic Control and Computers, University POLITEHNICA of Bucharest, 060042 Bucharest, Romania; alin.moldoveanu@cs.pub.ro (A.M.); irina.mocanu@cs.pub.ro (I.M.); florica.moldoveanu@cs.pub.ro (F.M.); emilian.radoi@cs.pub.ro (I.E.R.); victor.asavei@cs.pub.ro (V.A.); alex.gradinaru@cs.pub.ro (A.G.); 2 Faculty of Engineering, Lucian Blaga University of Sibiu, 550024 Sibiu, Romania; alex@butean.com

**Keywords:** indoor localization, computer vision, QR codes, fiducial markers, 3D reconstruction

## Abstract

Computer vision based indoor localization methods use either an infrastructure of static cameras to track mobile entities (e.g., people, robots) or cameras attached to the mobile entities. Methods in the first category employ object tracking, while the others map images from mobile cameras with images acquired during a configuration stage or extracted from 3D reconstructed models of the space. This paper offers an overview of the computer vision based indoor localization domain, presenting application areas, commercial tools, existing benchmarks, and other reviews. It provides a survey of indoor localization research solutions, proposing a new classification based on the configuration stage (use of known environment data), sensing devices, type of detected elements, and localization method. It groups 70 of the most recent and relevant image based indoor localization methods according to the proposed classification and discusses their advantages and drawbacks. It highlights localization methods that also offer orientation information, as this is required by an increasing number of applications of indoor localization (e.g., augmented reality).

## 1. Introduction

In recent years, the field of indoor localization has increased in popularity due to both the increasing number of applications [1] in domains such as surveillance [2], navigation (both assistive and general purpose) [3], robotics [4,5,6], and Augmented Reality (AR) [7] and the many proposed solutions that differ in terms of the devices used for tracking, the type of sensor data, and the localization algorithms.

**Figure 1:**
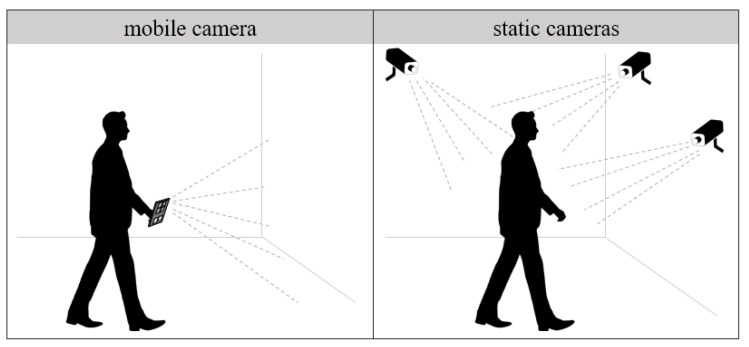
Indoor localization with a mobile camera (**left**) or with static cameras (**right**).

This paper focuses on computer vision based localization methods; therefore, the solutions presented are based on input from cameras. Most navigation systems use cameras carried by the subject, which represents the mobile entity (e.g., person, robot) that requires positioning or tracking, as illustrated in the left-hand side of Figure 1. The other type of solutions uses an infrastructure of static cameras positioned at known locations throughout the building to track the subject, as shown in the right-hand side of Figure 1. The vision based localization systems use 2D or 3D cameras (e.g., stereo, depth, RGB-D cameras) and perform the localization by identifying artificial markers (such as Quick Response (QR) codes and fiducial markers like AprilTags, ARTags, and CALTags [8]) or objects that are part of the environment. In many cases, the cameras are used in combination with other sensors such as WiFi, beacon, or inertial sensors [1].

Depending on the application, the required level of location accuracy varies [9]. Navigation solutions for guiding people to find specific rooms in a building or when changing underground lines accept an accuracy of several meters. In the same accuracy range are assistive solutions for the elderly that monitor their approximate location to confirm their compliance with certain routines and to detect situations of emergency such as a person ceasing to move. Other tracking or surveillance applications require 1–2 m accuracy to assess risky situations such as a person getting too close to an exhibit item in a museum. However, some of these surveillance applications require a higher accuracy of 10–20 cm when aiming to detect whether restricted areas/perimeters are only entered by authorized people. Applications for indoor autonomous robots or assistive systems for the visually impaired that perform obstacle detection cannot rely on an approximate localization and need centimeter-level accuracy. Modern AR solutions take the accuracy requirements even further. To offer seamless integration of the multimedia content, superimposed over video flows on smartphones or over smart glasses’ lenses, these applications require centimeter to millimeter accuracy of the position and orientation of the user’s mobile device.

Even though a considerable number of surveys on indoor localization have been published [1,4,5,6,9,10,11,12,13,14,15], as this research space continuously developed and the types of localization solutions diversified, we find that for the area of vision based localization, the majority of the previous surveys could have better focus as they are too general (encompassing all kinds of sensing devices) or too specific (addressing only a segment of the vision based localization problem, such as Simultaneous Localization and Mapping (SLAM) [16,17,18,19,20,21], Structure from Motion (SfM) [22], or image matching [23]). Other surveys discuss indoor positioning solutions particular to certain application domains. For instance, Huang et al. [24] analyzed only localization solutions that combined visual and inertial information. Marchand et al. [7] provided a survey of pose estimation methods used only for AR. Silva and Wimalaratne [25] presented a survey of navigation and positioning aids for the visually impaired. In comparison, we offer a comprehensive survey of image based localization solutions regardless of the application domain and propose a new classification.

The paper is organized as follows: the next section describes the most impacted domains by indoor localization; Section 3 classifies 70 selected computer vision based indoor localization methods and details their main characteristics; Section 4 focuses on benchmarks used for evaluating image based indoor localization solutions; and Section 5 presents our conclusions.

## 2. Application Domains

### 2.1. Assistive Devices

With the advance in technology, researchers have focused on improving the lifestyle of people with various disabilities, including visual impairment [26]. Two of their main problems are navigating and perceiving unknown environments.

There are several solutions to this problem that map the environment with images containing different visual cues such as QR codes, bar codes, or other simple synthesized geometric shapes like circles and triangles. Idrees et al. [27] proposed an indoor navigation system that used QR codes that were placed on the floor at certain locations. The system guided the user to a selected destination, checking the user’s location every time a QR code was scanned. Fusco and Coughlan [28] used sign detection and visual-inertial odometry to estimate the user’s location inside a building, requiring only a digital map of that building that contained the locations of the signs.

Other solutions create a 3D reconstruction of the environment in a configuration stage or create a database with images of the indoor space, annotated with location information. Endo et al. [29] proposed a navigation system for visually impaired people, which applied Large-Scale Direct SLAM (LSD-SLAM) to estimate the user’s position while constructing a 3D map of the environment. The 3D model of the environment allowed for the construction of an occupancy grid map, divided into quadrate cells, which stored information about the presence or absence of an obstacle in that location. The system created a cost map and conducted path finding through the navigation stack provided by the Robot Operating System (ROS) framework [30]. Li et al. [19] detected dynamic obstacles and applied path planning to improve navigation safety for people with visual disabilities. They built a 3D reconstruction of the environment with the visual positioning service provided by the Google Tango device. With a time-stamped map Kalman filter, they implemented an obstacle detection and avoidance algorithm that guided the user to a specified destination.

As previously mentioned, some localization solutions use an infrastructure of static cameras located at known positions in the environment. Heya et al. [31] employed color detection in an indoor localization system used for visually impaired people. A static camera located on the ceiling tracked the screen of a smartphone that was placed on the user’s shoulder. Chaccour and Badr [32] proposed an ambient navigation system that was composed of static cameras attached to the ceiling. The system detected the users’ location and orientation based on markers located on their heads. Static and dynamic obstacles were identified based on their shape or predefined images that were stored in a database. The proposed solution provided navigation assistance and obstacle avoidance and allowed the visually impaired users to locate missing objects.

Many navigation systems for visually impaired people assume the existence of a map for the current environment or create such a map in a configuration stage [25]. However, there are some solutions that allow the user to navigate in unknown environments by simply translating the visual information through audio or haptic signals, allowing the user to create a mental map of the surroundings. Sound of Vision [33,34] is such an example, which identifies the most important objects in the proximity of the user and sends information about their characteristics (weight, height, elevation, distance to the user, etc.) through headphones and a haptic belt. Sound of Vision is not only a navigation system, but also a solution for perceiving the environment. However, the understanding of the audio and haptic information that characterize the environment can be accomplished only through intensive training [35].

More information about indoor positioning systems for visually impaired people can be found in Siva’s and Wimalaratne’s survey [25].

### 2.2. Autonomous Robots

Designing autonomous robot applications can represent a challenge, since the localization methods cannot rely on external information. When navigating an environment, a human being can use the five senses, especially vision, touch, and hearing, to create a mental representation of the surroundings. This is not the case for robots, which navigate the environment only based on the information provided by the localization system. Therefore, localization solutions for autonomous robots require continuous computation of the robot’s position and orientation relative to a digital representation of the environment, as well as obstacle detection and path planning. On the other hand, designing applications for specific robots can simplify the localization problem. Various characteristics of a robot, such as degrees of freedom, width, height, position of the sensors mounted on the robot, or wheel diameter can be used to make assumptions about the movement of the robot (dead reckoning), thus reducing the complexity of the localization algorithms.

Since most robots operate in controlled environments, a popular approach is to configure the space with artificial landmarks such as QR codes. Li and Huang [18] presented a system that assisted robots, as well as human beings, in navigating indoor environments. A Kinect device acquired color information that allowed the detection of QR codes attached to the walls at known locations in a room. The depth sensor measured the distance from the Kinect camera to the identified QR codes. Babu and Markose [36] proposed a navigation system for Internet of Things (IoT) enabled robots. A QR code based detection solution estimated the position of the robot, while a path optimization step based on Dijkstra’s algorithm assisted the robot in reaching a destination node. Nazemzadeh and Macii [37] described a localization solution for unicycle-like wheeled robots. It computed the position of the robots by fusing information from QR codes, odometry based on dead reckoning, and a gyroscope platform. Cavanini et al. [38] proposed a low-cost QR code based localization system for robots operating indoors, experimentally validated on smart wheelchairs.

Other approaches used actual images of the environment, acquired with 2D or 3D cameras. Correa et al. [39] described a Kinect based reactive navigation system that guided robots while performing obstacle avoidance. It recognized different configurations of the indoor space with an artificial neural network. Xin et al. [40] introduced an RGB-D SLAM method that combined the Oriented FAST and Rotated BRIEF (ORB) and Random Sample Consensus (RANSAC) algorithms for feature extraction and matching. They created a 3D volumetric map of the environment that could be used for the navigation of a mobile robot. Kao and Huy [41] proposed an indoor navigation system that performed smartphone based visual SLAM with ORB features using a wheel-robot. They combined WiFi signals, information from inertial sensors, and monocular images for the computation of the robot’s position.

Surveys dedicated to positioning solutions for autonomous robots [4,5] present in-depth information on this topic.

### 2.3. Augmented Reality

Currently, Augmented Reality (AR) has become very popular, due to the new technologies that are bringing it closer to the greater public. Smartphones are the most commonly used devices for displaying augmented content. However, smart glasses, such as Moverio [42] or Google Glass [43], are gaining ground [44]. For a seamless integration of the multimedia content within the real environment, the position and orientation of the display device must be estimated with high accuracy.

Gerstweiller [45] presented HyMoTrack , a tracking solution that generated a 3D model of the environment out of a vectorized 2D floor plan, and an AR path concept, called FOVPath, for guiding people. Using the FOVPath approach, the display of a trajectory depended on the user’s position and orientation and also on the Field Of View (FOV) capabilities of the device. Wang et al. [46] described the development of a 3D augmented reality mobile navigation system that provided indoor localization based on Radio-Frequency Identification (RFID) readings and computer vision. They created 3D representations of internal and external structures of Oxford College, from the present and the past. Based on the position and orientation of the user’s device, they displayed the 3D architectural appearance of the college during important time periods, as well as multimedia content such as texts, pictures, or 3D models related to various exhibits. Balint et al. [47] presented an AR multiplayer treasure hunt game, which combined GPS position information with localization based on image recognition. The treasures were virtual 3D objects that were displayed when the user reached a checkpoint, and the device was oriented towards the respective direction. Baek et al. [48] proposed an AR system for facility management, which computed the user’s pose relative to the building, with a deep learning approach. The visualization module displayed location-specific information, holographic pipes in this case, which in reality were not visible because they were built within the walls.

Marchand et al. [7] offered more information on the topic of pose estimation for augmented reality, presenting the most important approaches on vision based positioning.

#### AR Commercial Solutions

This section presents some of the most popular commercial tools for developing augmented reality content, which have indoor localization capabilities.

Wikitude [49] is an augmented reality Software Development Kit (SDK) that uses the SLAM technology to reconstruct the environment. It also performs 2D and 3D image recognition and tracking, which can trigger the display of digital content, overlaid on the real world.

ARKit [50] is an iOS AR platform that provides scene understanding capabilities by combining inertial data with visual information to detect horizontal and vertical planes. It also recognizes images and 3D objects, determining the position and orientation of the camera relative to the target.

ARCore [51] is another SDK, launched by Google, which allows developers to build augmented reality experiences that seamlessly integrate the digital content into the real world. ARCore provides motion tracking capabilities, as well as environmental understanding based on plane detection. It performs SLAM, making use of inertial sensors and the data acquired with a smartphone camera, estimating the position and orientation of the user’s device relative to a 3D coordinate system.

Vuforia [52] is a popular engine that provides detection and tracking of image targets and pose estimation of any tracked target or marker, allowing the rendering module to display the 3D virtual content naturally, depending on the position and orientation of the user. The pose computation is performed only relative to an image target or a marker, therefore not offering actual indoor positioning capabilities (relative to an entire room or another type of indoor space).

ARToolKit [53] is an open-source library intended for the development of augmented reality applications, which overlays 2D and 3D multimedia content on the real world. It is a tracking library that computes the camera position and orientation relative to square markers or to natural feature markers in real time. It works with both monocular and stereo cameras, providing calibration capabilities.

MAXST [54] is a cross-platform engine that provides a variety of tracking features for the development of augmented reality applications. It recognizes and tracks planar target images or planar surfaces, as well as markers with regular patterns or QR codes. Their implementation of visual SLAM can be used to create map files of the environment that are later loaded up by the Object Tracker module, which superimposes AR experiences on them. Another module, AR Fusion tracker, generates world representations and performs environment tracking by combining information from the other tracking modules.

Other popular commercial solutions for developing augmented reality applications are EasyAR [55], Kudan [56], Onirix [57], Pikkart [58], and DeepAR [59].

### 2.4. Surveillance and Monitoring

Indoor positioning can also be used for surveillance or monitoring purposes, detecting whether an unauthorized person has breached a perimeter, or tracking a certain person throughout an entire building or house. Generally, surveillance systems use an infrastructure of static cameras for the purpose of detecting and tracking the users. However, there are cases when information from other sensors, such as WiFi access points or beacons, is fused with the video frames.

Sun et al. [60] proposed a localization solution that used panoramic cameras and a map of the indoor environment. They applied a background subtraction method to detect human beings, matching their location to a corresponding position on the indoor map.

Desai and Rattan [61] used a pan/tilt camera and wireless sensor networks to track objects within an indoor space. The estimation of an object’s position was performed with the time difference of arrival method. The camera, equipped with a laser pointer, followed the object continuously, by computing the pan and tilt angles based on a listener Cricket mote carried by the object. Grzechca et al. [62] fused Received Signal Strength Indication (RSSI) information with data from a static video camera to track human beings in indoor environments. Zhang et al. [63] acquired video sequences with a surveillance camera and recognized a target person by matching the information provided by the inertial sensor of the person’s smartphone with gait and heading azimuth features extracted from the videos. They applied a Convolutional Neural Networks (CNN) based object tracking technique in order to handle occlusion.

As far as we know, there is no recent survey on computer vision based indoor localization dedicated to surveillance and monitoring, but further information on this topic can be found in the work of Shit et al. [64], which presented localization solutions with static cameras, and in the survey of Jiao et al. [65], which discussed deep learning methods for object positioning.

## 3. Indoor Localization Solutions

### 3.1. Selection of Papers Included in the Survey

**Figure 2:**
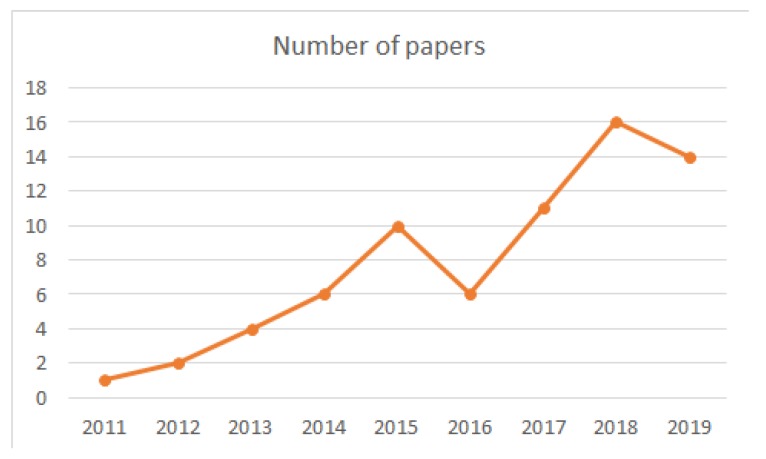
Distribution of selected papers over time. The horizontal axis shows the publication years. The vertical axis shows the number of papers published per year.

Image based indoor localization has been intensely researched. Among existing scientific publications, we chose 70 papers based on publication date and relevance to the domain, using only prestigious research databases (IEEE Explore, ACMDigital Library, SpringerLink, MDPI, and Elsevier). Figure 2 shows the distribution of selected papers over time, illustrating an increased interest in the image based localization domain in the last five years. Since it takes time for research papers to acquire visibility, the number of citations was not one of the selection criteria, as it disadvantaged the more recent research. The purpose of this survey was not to be exhaustive in terms of listing the work performed in the field of vision based indoor positioning, but to illustrate the main characteristics of the existing technologies and techniques. This allows the reader to attain an overview of the domain while understanding the advantages and drawbacks of the various methods. Choosing an appropriate solution does not boil down to just the application domain, but also to the particular requirements of the applications such as accuracy, computing time, equipment, and dynamic and static aspects (the properties of the objects contained in the environment).

### 3.2. Classification

Recent surveys in the indoor positioning domain have proposed various classifications. For instance, the survey of Yassin et al. [1], which addressed the entire domain of indoor localization (not limited to vision based solutions), proposed a two-level classification. The first level grouped the solutions based on the positioning algorithms, which were divided into three classes: triangulation, scene analysis, and proximity detection. The second level classified the solutions within the first level classes based on the measurement techniques as follows: the triangulation class had two sub-classes: lateration and angulation; the scene analysis class had only one sub-class: fingerprinting based; and the proximity detection class had two sub-classes: cell-ID and RFID. Another general survey in indoor localization [10] classified existing research solutions into local infrastructure dependent techniques (ultra-wideband, wireless beacons), local infrastructure independent techniques (ultrasound, assisted global navigation satellite systems, magnetic localization, inertial navigation systems, visual localization, infrared localization), and visual/depth sensors (structured light technology, pulsed light technology, stereo cameras).

Mendoza-Silva et al. [9] presented a meta-review of indoor positioning systems, resulting from the analysis of 62 indoor localization-related surveys. They reviewed the most commonly used technologies for localization applications and proposed the following classes: light, computer vision, sound, magnetic fields, dead reckoning, ultra-wideband, WiFi, Bluetooth Low Energy, RFID, and Near-Field Communication (NFC). In the computer vision class, they discussed several positioning techniques, such as visual odometry and vision based SLAM, and mentioned different acquisition devices (monocular, stereo, omnidirectional). However, they did not propose any classification for this domain. They also observed the complete lack of recent surveys on computer vision based indoor localization solutions and claimed the necessity of such a work.

**Figure 3:**
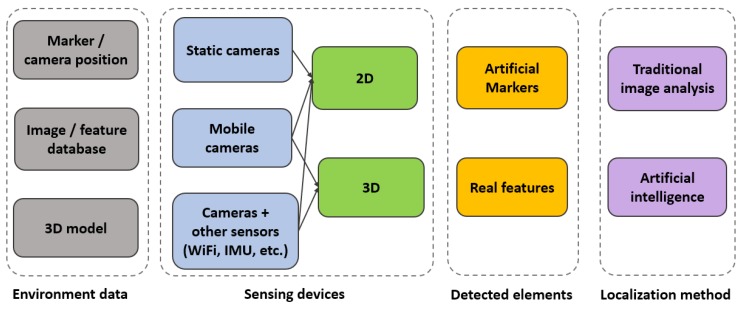
Proposed classification based on environment data, sensing devices, detected elements, and localization method.

Analyzing the research papers mentioned in the previous section, several discriminating characteristics emerged. Therefore, we propose a new classification of computer vision based indoor localization solutions, as illustrated in Figure 3.

All indoor positioning methods have a configuration stage, in which the environment is filled with landmarks and sensors, images from the environment are saved into a database, or a 3D representation of the indoor space is created. Therefore, environment data could consist of information about the position of the markers (e.g., QR codes, geometric synthetic identifiers) or the location of the static cameras placed within the scene. Another type of environment data is represented by databases with images or features from images, annotated with position and orientation information. Lastly, environment data could consist of a 3D model of the environment, a point cloud, a 3D mesh, or a 3D map, obtained with various methods such as manual modeling, SLAM, or SfM.

Another element that helps discriminate between methods is the type of employed sensing devices. As previously mentioned in Section 1 and illustrated in Figure 1, the main acquisition devices are static and mobile cameras. Furthermore, the input information can be enriched with data from other sensors, such as WiFi access points or IMU devices. Another differentiating aspect of image based localization methods is the type of visual input, which can be either 2D or 3D.

The localization methods can search for artificial markers (e.g., QR codes and other fiducial markers such as AprilTags [66], ARTags, and CALTags [8]) or for features from the real environment. The latter category includes any type of element that can be extracted from the real environment (without the need to insert synthesized items into the scene), either features of interest such as Speeded Up Robust Features (SURF) and Scale-Invariant Feature Transform (SIFT) or semantic objects. Therefore, we propose a new level of classification, namely the *detected elements*, which refers to the type of features (artificial markers or natural, real elements from the environment) that are tracked or matched within the images.

Indoor positioning solutions employ various localization methods, which range from low-level feature matching to complex scene understanding. We divided the techniques into traditional image analysis and artificial intelligence. The ones belonging to the second category include any type of artificial intelligence, such as Bayesian approaches, Support-Vector Machine (SVM), and neural networks.

We applied the proposed classification to the selected indoor localization solutions. Table 1 assigns each of the chosen research papers to a class, based on environment data, sensing devices, detected elements, and localization method.

Out of all the classes that could result from combining the differentiating elements from Figure 3, we chose only 17 of the more popular ones, which were represented by a large number of research papers.

The following sub-sections analyze each category, presenting representative indoor localization solutions and discussing their advantages and drawbacks. For each examined scientific paper, we include in Table 2, Table 3, Table 4, Table 5, Table 6, Table 7, Table 8, Table 9, Table 10, Table 11, Table 12, Table 13, Table 14, Table 15, Table 16, Table 17 and Table 18 information about the characteristics of the datasets used for evaluation, the computing time or refresh rate (related to a certain running platform), and the achieved accuracy. If there were papers that did not report information about a certain characteristic, the field corresponding to that characteristic is marked with “-”. Some papers evaluated their solutions only visually, while others applied various metrics, such as average and/or absolute errors for position and orientation, percentage of tested cases when accuracy was within certain intervals, Detection Success Rate (DSR), Root Mean Squared Error (RMSE), Navigation Success Rate (NSR), Relative Pose Error (RPE), and Absolute Trajectory Error (ATE).

#### 3.2.1. Indoor Localization Solutions with 2D Static Cameras, Markers, and Traditional Image Analysis

This class of indoor localization methods uses an infrastructure of 2D static cameras with known locations. The images from these cameras are processed with traditional computer vision algorithms in order to detect synthetic identifiers carried by people or robots.

Belonging to this class is the work of Heya et al. [31], where the screen of the user’s smartphone was detected with a simple color tracking algorithm. Each user was assigned a color, which was displayed on the smartphone, and the system tracked the screen of the device, which was placed on the user’s shoulder. Another example of indoor localization solution using static 2D cameras and traditional image processing was an ambient navigation system proposed by Chaccour and Badr [32], which detected the users’ location and orientation based on markers located on their heads. The system was evaluated within a home composed of three rooms, kitchen, living room, and bedroom, each containing an IP camera placed on the ceiling. The tests performed with eight people, including dynamically added obstacles, proved the reliability of the system.

The methods in this class require the map of the building and a configuration step that consists of annotating the positions of the static cameras on the map. They can achieve good, centimeter-level, accuracy, as can be seen in Table 2, which makes them viable solutions for scenarios requiring high accuracy positioning in small spaces. However, maintaining this accuracy level in large indoor spaces comes with high costs in terms of both effort and infrastructure, due the cumbersome configurations and the high number of cameras required.

#### 3.2.2. Indoor Localization Solutions with 2D Static Cameras, Real Features, and Traditional Image Analysis

In this class of indoor localization solutions, the images from the static cameras are processed with traditional computer vision algorithms in order to track objects or people and compute their positions within a certain room. Localization solutions within this class identify people or robots without the need for the tracked entities to carry devices or artificial markers.

Bo et al. [67] recursively updated the position of multiple people based on the detected foreground and the previous known locations of each person. The foreground was identified by analyzing changes in image structure (edges) based on the computation of the normalized cross-correlation for each pixel. They applied a greedy algorithm to maximize the likelihood of observing the foreground for all people. The efficiency of their algorithm was evaluated on public datasets, using the Multiple Object Tracking Accuracy (MOTA), a metric computed based on object misses, false positives, and mismatches.

Shim and Cho [69] employed a homography technique to create a 2D map with accurate object position, using several surveillance cameras. Dias and Jorge [68] tracked people using multiple cameras and a two level processing strategy. Firstly they applied region extraction and matching to track people, and secondly, they fused the trajectories detected from multiple cameras in order to obtain the positions relative to a global coordinate system, using homography transformations between image planes.

Sun et al. [60] proposed a device-free human localization method using a panoramic camera. They employed pre-processing, human detection with background subtraction (with mean filtering and a Gaussian low pass filtering), and an association between the location of users in the image space and their location on a given map of the indoor environment.

Compared to the previous class of solutions that use artificial markers, the methods in this class have slightly higher localization errors, as can be observed in Table 3. However, this accuracy level (tens of centimeters) is still good for many types of applications, and these methods have a wider applicability, especially in the monitoring and surveillance domains, due to them not requiring the tracked entities to carry devices or markers.

#### 3.2.3. Indoor Localization Solutions with 2D Static Cameras, Real Features, and Artificial Intelligence

This class of indoor localization methods differs from the class described in Section 3.2.2 by the type of employed algorithms for determining the entities’ positions. An alternative to traditional image processing algorithms is artificial intelligence, in the form of Bayesian approaches, SVM, or neural networks. For instance, Utasi and Benedek [70] proposed a Bayesian method for people localization in multi-camera systems. First, pixel-level features were extracted, providing information about the head and leg positions of pedestrians. Next, features from multiple camera views were fused to compute the location and the height of people with a 3D Marked Point Process (MPP) model, which followed a Bayesian approach. They evaluated their method on two public datasets and used the Ground Position Error (GPE) and Projected Position Error (PPE) metrics for accuracy computation. Cosma et al. [73] described a location estimation solution based on 2D images from static surveillance cameras, which used pose estimation from key body points’ detection to extend the pedestrian skeleton in case of occlusion. It achieved a location estimation accuracy of approximately 45 cm, as can be observed in Table 4, in complex scenarios with a high level of occlusion, using a power efficient embedded computing device. See-your-room [74] represents another localization solution that uses cameras placed on the ceiling. It employs Mask R-CNN and OpenPose [128] to detect people and their pose (standing, sitting) and the perspective transformation to obtain the position of the users on a map. Hoyer et al. [71] presented a localization framework for robots based on Convolutional Neural Networks (CNN) using static cameras. In a first stage, they used a CNN object detection to estimate the type and the bounding box of a robot. In the second stage, they ran two more neural networks, one for computing the orientation of the robot and another one to provide identification (based on a code placed on the robot). An algorithm was also proposed for generating synthetic training data by placing contour-cropped images of robots on background images. The solution described by Jain et al. [72] was based on the assumption that, in an office, employees tend to keep their phones lying on the table and that the ceiling layout is unique throughout the building, containing different tiles. They used a combination of artificial intelligence and traditional image processing to detect landmarks such as ceiling tiles, heating or air conditioning vents, lights, sprinklers, audio speakers, or smoke detector sensors. First, they applied the Hough transform to extract tiles, then SURF for feature extraction, and SVM to classify the type of landmark with the ECOCframework [129].

Table 4 presents the characteristics of the localization methods that use 2D static cameras, real features, and artificial intelligence based algorithms. The computational challenge of using neural networks or other AI based implementations can be met with the use of GPUs, as can be observed for several methods [71,73], which achieve interactive or real-time performance. Although a higher complexity of the algorithms would lead to expecting a higher accuracy level compared to the previous class of solutions, relevant accuracy comparisons cannot be made due to the evaluations being performed on different datasets/scenarios.

#### 3.2.4. Indoor Localization Solutions with 2D Mobile Cameras, Markers with Known Positions, and Traditional Image Analysis

This class of indoor localization solutions employs a configuration step, in which artificial landmarks, predominantly QR codes, are placed at known locations inside a building (generally on the ceiling, walls, or floor). These solutions make use of cameras attached to people or robots and apply traditional image processing during the localization stage. Each QR image codifies its position within the coordinate system of the building. Based on the appearance of the QR code in the acquired images during the localization stage, compared to the raw images of the QR codes, the orientation of the camera can also be estimated by computing the projective transform matrices.

QR codes allow for fast detection and decoding of stored information. However, in cases where the video camera is moving fast, the detection of these codes can be difficult. This led Lee et al. [75] and Goronzy et al. [76] to surround their codes with simple borders such as circles or rectangles, which can be detected faster than QR codes with Hough transform.

Ooi et al. [79] used QR codes to reposition mobile sensor networks, in the form of four wheeled robots. When QR codes were not in range, the system estimated the position of the robot using dead reckoning.

Lightbody et al. [78] proposed WhyCode, a new family of circular markers that enable faster detection and pose estimation, of up to two orders of magnitude compared to other popular fiducial marker based solutions. They extended the WhyCon algorithm [130], which localizes a large number of concentric black and white circles with adaptive thresholding, flood fill, and a circularity test. The position of a marker, along with the pitch and roll, was estimated based on eigenvalues with a method proposed by Yang et al. [131]. The yaw was computed by detecting the Necklace code contained in the WhyCode marker. Benligiray et al. [80] presented STag, a fiducial marker system that used geometric features to provide stable position estimation. The markers contained an inner circular border and an outer square border used for detection and homography estimation. They compared their detection capabilities against the ARToolkit, ArUco [132], and RUNE-Tag [133] fiducial markers. Khan et al. [81] proposed a generic approach for indoor navigation and pathfinding using simple markers (ARToolkit) printed on paper and placed on ceilings. The orientation of the smartphone relative to a marker enabled the computation of the user’s direction along a certain path.

As can be observed in Table 5, the performance of these methods is quite impressive. The centimeter or even sub-centimeter level position accuracy is achieved due to the precise matching mechanism when dealing with synthesized images. The fast detection and decoding of QR codes and fiducial markers enables real-time applications.

Compared to the previously presented static camera based solutions, even though deploying such a system in a large built environment also comes with a considerable effort in the configuration stage, it is significantly less expensive (artificial markers are practically free in comparison to static cameras). However, the tracked entity is required to carry a mobile camera, which in certain scenarios can represent an inconvenience, and mapping a building with artificial images can have a negative impact on the building’s appearance.

Another important aspect when choosing marker based localization solutions is their detection success when facing occlusion. This problem was addressed in the solution proposed by Garrido-Jurado et al. [132], which combined multiple markers with an occlusion mask computed by color segmentation. Sagitov et al. [8] compared three fiducial marker systems, ARTag, AprilTag, and CALTag, in the presence of occlusion, claiming that CALTags showed a significantly higher resistance for both systematic and arbitrary occlusions.

#### 3.2.5. Indoor Localization Solutions with 3D Mobile Cameras, Markers with Known Positions, and Traditional Image Analysis

Localization based on fiducial markers can also be performed by analyzing RGB-D images with traditional image processing methods. Li et al. [82] used RGB-D images in order to detect and recognize QR landmarks with the Zbar [135] code reader. The distance to the QR code was computed based on the depth image. Dutta [83] proposed a real-time application for localization using QR codes from RGB-D images, based on the keystone effect in images from range cameras (the apparent distortion of an image caused by projecting it onto an angled surface).

Some solutions achieve centimeter accuracy when computing the distance from the camera to the artificial marker (see Table 6). These solutions are very practical, since 3D cameras already offer a depth map of the environment, allowing for a faster and less complex computation of the position in a 3D coordinate system. However, as can be observed in Section 3.2.4, detection and pose computation for markers is very fast for 2D cameras as well, due to the geometric properties of the synthetic images. Therefore, using 3D cameras could represent an unnecessary excess of resources. Furthermore, RGB-D cameras usually have a lower resolution than RGB cameras, both for the color and depth maps. Thus, their use is rarely justified for marker based solutions.

#### 3.2.6. Indoor Localization Solutions with 2D Cameras + Other Sensors, Markers with Known Positions, and Traditional Image Analysis

Synthetic identifiers represent a very powerful tool when estimating the subject’s position and orientation in indoor scenarios. However, the use of other sensors, such as inertial sensors, WiFi, or beacons, could enrich the information, thus increasing the accuracy, or could help reduce the number of necessary synthetic landmarks. Nazemzadeh et al. [37] proposed a localization solution for unicycle-like wheeled robots, using Zbar and OpenCV to detect QR codes that were placed on the floor. They applied an Extended H-Infinity Filter (EHF) to compute the odometry based on dead reckoning and on a gyroscope platform. Babu and Markose [36] also invoked dead reckoning with accelerometer and gyroscope information, increasing the accuracy of their QR based localization solution.

Gang and Pyun [84] configured the indoor space, in an offline phase, by creating a fingerprint map with the RSSI of the beacon signals and the intensity of the geomagnetic field at each reference point. In the localization stage, they combined the information from the beacons and the inertial sensors with the coordinates extracted from QR codes, obtaining an accuracy of approximately 2 m, as can be observed in Table 7.

The use of other sensors besides cameras can add many benefits to a localization solution, especially if there is no need to acquire supplementary equipment. This is the case for WiFi access points, already installed in a building for other purposes. However, most of the WiFi localization solutions are based on the WiFi fingerprinting procedure, a manual and cumbersome configuration stage in which the signal strengths of the access points are recorded for known locations on the map of the building.

Since smartphones have become very popular and their cameras have reached impressive capabilities, they can be successfully used as acquisition devices in computer vision based localization solutions. Another advantage of using a smartphone is represented by the built-in inertial sensors. Thus, an application that combines input from the camera and the inertial sensors of a smartphone does not require equipment that is not already owned by the users.

#### 3.2.7. Indoor Localization Solutions with Real Image/Feature Databases, 2D Mobile Cameras, and Traditional Image Analysis

Using a database of real images or features from real images of the environment in localization solutions represents an alternative to decorating the indoor space with QR codes or other synthesized images.

In a configuration stage, images or features, labeled with location and orientation information, are stored in a database. For instance, Hu et al. [85] obtained a panoramic video of the scene, which was processed with traditional computer vision algorithms for computing omni-projection curves. Bai et al. [86] constructed a landmark database by using a laser distance meter to measure the distance between the location of the camera and selected landmarks.

In the localization stage, the images acquired with the mobile camera were compared with the ones from the database using feature matching algorithms such as SIFT, SURF, or ORB. The processing time in this stage is highly affected by the number of images/features in the database, which must be compared against the images from the mobile camera’s video flow. The first line of Table 8 is a good example, as it shows that running the localization algorithm with a database of 1000 frames was eight times faster than with a database of 8000 frames. To reduce the processing time, Elloumi et al. [87] limited the similarity search of two images to only a selection of areas within the images, thus reducing the number of features by 40%. These areas were considered to contain the most important characteristics and were selected based on a metric that combined orientation, color, intensity, flickering effects, and motion.

Compared to solutions that use artificial markers, the solutions in this class do not require decorating the indoor space with visual markers, thus not affecting the aesthetics of the indoor space. Although they have a higher localization error (few meters), this error level can still be acceptable for certain applications.

#### 3.2.8. Indoor Localization Solutions with Real Image/Feature Databases, 2D Mobile Cameras, and Artificial Intelligence

Artificial intelligence includes a plethora of localization algorithms for systems that use mobile cameras. For instance, Lu et al. [89] proposed a multi-view regression model to determine the location and orientation of the user accurately. Xiao et al. [90] determined the location of a smartphone, based on the detection of static objects within images acquired with the smartphone’s cameras. Faster-RCNN was used for static object detection and identification. Another deep CNN, Convnet, was used in the localization system proposed by Akal et al. [91]. This network uses compound images from four non-overlapping monocular images placed on a ground robot, achieving centimeter accuracy, but requiring a sizeable dataset of compound images for training. As can be observed in Table 9, the machine learning based solutions achieved interactive computing times or even real-time performance and a localization accuracy of under one meter to tens of centimeters. These solutions seemed to have better accuracy performance compared to the solutions in the previous class, while benefiting from the same advantages of not requiring deploying visual markers in the indoor space.

#### 3.2.9. Indoor Localization Solutions with Real Image/Feature Databases, 3D Mobile Cameras, and Artificial Intelligence

Another class of indoor localization methods uses RGB-D images acquired with mobile cameras that are processed with the help of CNN. Guo et al. [92] used a CNN (PoseNet network) for exploiting the vision information and the long short-term memory network for incorporating the temporal information. Zhang et al. [63] applied visual semantic information for performing indoor localization. A database with object information was constructed using Mask-RCNN, extracting the category and position for each object. Then, using the SURF descriptor, keypoints of the recognized objects were detected. Furthermore, CNN features were obtained using a pre-trained ResNet50 network. The visual localization was performed in two steps: the most similar key frames were obtained using the selected CNN features; the bundle adjustment method [137] was used to estimate the matrix between the current image and candidate frames. Both methods were tested on public datasets. Localization results were within 0.3 m and 0.51 m (as shown in Table 10).

3D cameras give access to a depth map of the environment, either through built-in algorithms, as in the case of structured light or time-of-flight devices, or through stereo matching algorithms that have multiple implementations, available to the public. However, these cameras come with various limitations. For instance, the estimation of the depth map with stereo cameras in the case of untextured surfaces (such as white walls) is very inaccurate. Furthermore, structured light and time-of-flight depth cameras cannot estimate the distance to reflective surfaces or in case of sunlit environments. Moreover, although 3D cameras have gained popularity, they are not as common as 2D cameras, and therefore, their applicability is reduced. While localization solutions with 2D mobile cameras can be easily deployed, using generally available smartphones, 3D cameras are more appropriate for specialized applications, in areas like assistive devices or autonomous robots.

#### 3.2.10. Indoor Localization Solutions with Real Image/Feature Databases, 2D Cameras + Other Sensors, and Traditional Image Analysis

If WiFi signals, inertial sensors, beacons, or other sensors can increase the accuracy of marker based localization solutions or can help reduce the number of synthesized images that should be placed on the ceiling/floor/walls of the building (as discussed in Section 3.2.6), a hybrid approach can be even more useful when dealing with natural features from the environment. Acquiring additional information from various sensors can help reduce the search space in the image matching stages.

Yan et al. [94] also used WiFi information to increase the accuracy and improve the processing time of a natural feature extraction algorithm, which combined Features from Accelerated Segment Test (FAST) with SURF.

Marouane et al. [93] used accelerometer data for step counting and gyroscope information for orientation and transformation of images into histograms for more efficient image matching. Rotation invariance was achieved by adding the perspective transformation of two planes. Another solution that used inertial sensors was the one proposed by Huang et al. [95]. They applied the vanishing points method and indoor geometric reasoning, taking advantage of rules for 3D features, such as the ratio between width and height, the orientation, and the distribution on the 2D floor map. Arvai and Dobos [96] applied the perspective-n-point algorithm to estimate the user’s position inside the 2D floor-plan of a building, relative to a series of landmarks that were placed in the configuration stage. They used an extended Kalman filter to estimate the position by combining visual and inertial information.

Table 11 presents the characteristics of indoor localization solutions that combine data from 2D cameras and other sensors, estimating the position and orientation of the subject with traditional image processing. Several such solutions achieved centimeter location accuracy, due to this fusion between images and information from inertial sensors, WiFi signals, RFID devices, or beacons. However, this fusion of data from several sensors brings a computational load.

#### 3.2.11. Indoor Localization Solutions with Real Image/Feature Databases, 2D Cameras + Other Sensors, and Artificial Intelligence

The solutions based on the detection of objects or markers from RGB images offer a relative position and orientation estimation, but are unreliable when markers or objects are not visible. Furthermore, detection is influenced by camera exposure time. Thus, images combined with data from other sensors can increase the precision of the localization.

Rituerto et al. [97] estimated the user’s location using values acquired from inertial sensors combined with computer vision methods applied on RGB images. The particle filtering method was used for combining all these data. A map with walls, corridors, and rooms and some important signs (such as exit signs and fiducial markers) was also considered.

Neges et al. [98] combined an IMU step based counter with video images for performing indoor localization. IMU data were used to estimate the position and orientation of the mobile device, and different semantic objects were extracted from the video (e.g., exit signs, fire extinguishers, etc.) for validation of the obtained position. The recognition of different markers was achieved using Metaio SDK [140], a machine learning based development tool. In Sun et al. [99], RSS samples, surveillance images, and room map information were used for performing indoor localization. People were detected using background subtraction from images acquired with a camera placed on the ceiling of the room. The foreground pixel that was the nearest to the location of the camera would approximate the person position in the image. Then, this position was mapped to a localization coordinate using a multi-layer neural network (with three layers). The iStart system [100] combines WiFi fingerprints and RGB images for indoor localization. The system proposed by Zhao et al. [101] was based on a combination of CNN with a dual-factor enhanced variational Bayes adaptive Kalman filter. Channel State Information (CSI) was extracted from an MIMO-OFDM PHY layer as a fingerprint image to express the spatial and temporal features of the WiFi signal. CSI features were learned with a CNN inspired by the AlexNet network obtaining the mapping relationship between the CSI and the 2D coordinates. Results were processed with the Bayes adaptive Kalman filter in order to achieve noise attenuation. These methods were evaluated on their own datasets with good results (position accuracy of approximately 1 m), as shown in Table 12.

Even though artificial intelligence and especially deep convolutional networks have become very popular, they still come with certain limitations. First, they require a large amount of training data, usually manually annotated. Second, the training stage is both time consuming and hardware demanding. Even though in the online stage, the already trained network requires less resources, adding the complexity of fusing the visual data with information from other sensors can have a negative impact on the runtime, as can be observed for several selected papers [97,100].

#### 3.2.12. Indoor Localization Solutions with Real Image/Feature Databases, 3D Mobile Cameras + Other Sensors, and Traditional Image Analysis

Localization precision can be increased by matching of RGB-D images using traditional feature descriptors combined with information obtained from an IMU sensor. In Gao et al. [102], key points were extracted from the RGB-D images using an improved SIFT descriptor. Then, the RANSAC algorithm [141] eliminated mismatched points from the matching pairs. Their corresponding depth coordinates were obtained from the depth images. Using this information, the rotation matrix and translation vector were computed from two consecutive frames. Furthermore, IMU data were used to eliminate the noise, improving the stability and positioning accuracy. Adaptive fading extended Kalman filter fused the position information of Kinect and IMU outputs. Furthermore, this fusion eliminated the noise and improved the stability and accuracy of the system. A similar idea was proposed by Kim et al. [103]. Their solution generated 3D feature points using the SURF descriptor, which were next rotated using IMU data to have the same rigid body rotation component between two consecutive images. The RANSAC algorithm [141] was used for computing the rigid body transformation matrix. Table 13 shows the dataset characteristics and obtained accuracy for the localization methods based on RGB-D images processed with traditional image analysis algorithms and sensor fusion. Since robots can be equipped with many sensors, including 3D cameras and inertial units, the solutions in this class have been successfully applied to the autonomous robots domain.

#### 3.2.13. Indoor Localization Solutions with a 3D Model of the Environment, 2D Mobile Cameras, Real Features, and Traditional Image Analysis

Simultaneous Localization and Mapping is a very popular algorithm in several domains, such as autonomous robots or Augmented Reality. During recent years, various solutions to the problem of localization and mapping have been proposed. For instance, Endo et al. [29] used LSD-SLAM for map construction, localization, and detection of obstacles in real time. Teixeira et al. [104] used the pattern recognition SURF method to locate natural markers and reinitialize Davison’s Visual SLAM [142].

Several SLAM based solution use 3D cameras in the configuration stage, to create a 3D reconstruction of the environment, and then change the acquisition device to a monocular camera in the localization stage. Sinha et al. [105] applied RGBD-SLAM on images acquired with Microsoft Kinect to reconstruct 3D maps of indoor scenes. In the localization stage, they used monocular images acquired with a smartphone camera and estimated the transformation matrix between frames using RANSAC on the feature correspondences. They applied SIFT or SURF for feature extraction, in order to detect landmarks, which were cataloged as sets of distinguished features regularly observed in the mapping environment, being stationary, distinctive, repeatable, and robust against noise and lighting conditions. Deretey et al. [106] also applied RGBD-SLAM in an offline, configuration stage, to create 3D point clouds that contained intensity information. 2D features were extracted with a matching algorithm (SIFT, SURF or ORB), and then, a projection matrix of matched features between 2D images and 3D points was computed. A comparison with RGBD-SLAM was offered by Zhao et al. [109], which used Kinect to collect the 3D environment information in a configuration stage. They also built a 2D map of the indoor scene with Gmapping, an ROS package that used Rao–Blackwellized Particle Filters (RBPF) [143] to learn grid maps. In the online phase, they applied Monte Carlo localization based on the previously created 2D map.

Ruotsalainen et al. [107] performed Visual SLAM for tactical situational awareness by applying a Kalman filter to combine a visual gyroscope and a visual odometer. The visual gyroscope estimated the position and orientation of the camera by detecting straight lines in three orthogonal directions. The visual odometer computed the transformation of the camera from the motion of image points matched using SIFT in adjacent images. A similar approach, which took into account the structural regularity of man-made building environments and detected structure lines along dominant directions, was the solution proposed by Zhou et al. [108]. They also applied an extended Kalman filter to solve the SLAM problem. Ramesh et al. [110] combined imaging geometry, visual odometry, object detection with aggregate channel features, and distance-depth estimation algorithms into a Visual SLAM based navigation system for the visually impaired.

A different approach was the one proposed by Dong et al. [111], which reused a previous traveler’s (leader) trace experience to navigate future users or followers. They used ORB features for the mobile Visual SLAM. To combat environmental changes, they culled non-rigid contexts and kept only the static contents in use.

SLAM based approaches can attain centimeter or even millimeter location accuracy, but at a high computational cost. They also require significant memory resources to store the 3D representation of the scene. Table 14 presents the characteristics of some solutions that create a 3D reconstruction of the environment in an offline stage, acquire images with a monocular camera in the localization stage, and perform low-level image processing to estimate the position and orientation of the user/robot.

#### 3.2.14. Indoor Localization Solutions with a 3D Model of the Environment, 2D Mobile Cameras, Real Features, and Artificial Intelligence

Artificial intelligence based 2D localization methods can also be applied on 3D representations of the space. Han et al. [112] removed obstacles detected with the Mask-RCNN network to enhance the performance of the localization. It detected persons as potential obstacles and split these obstacles from the background. Then, ORB-SLAM2 [148] was used for localization. Xiao et al. [113] proposed Dynamic-SLAM for solving SLAM in dynamic environments. It was based on ORB-SLAM. First, a CNN was used for static or dynamic object detection. Then, applying a missed detection compensation algorithm based on the speed invariance from adjacent frames, the detection recall rate was improved. Finally, tracking was performed using ORB features extracted from each keyframe image for performing feature based visual SLAM by processing feature points of dynamic objects. The pose estimation was obtained by solving the perspective-n-point problem with the bundle adjustment method.

Table 15 presents the characteristics of some solutions belonging to the current class. The neural networks introduce a high computational load, but can help not only with the localization, but also with the scene understanding problem.

#### 3.2.15. Indoor Localization Solutions with a 3D Model of the Environment, 3D Mobile Cameras, Real Features, and Traditional Image Analysis

Several localization solutions use 3D cameras in the configuration step, as well as in the actual localization stage. For instance, Du et al. [114] created an interactive mapping system that partitioned the registration of RGB-D frames into local alignment, based on visual odometry, and global alignments, using loop closure information to produce globally consistent camera poses and maps. They combined RANSAC inlier count with visibility conflict in the three point matching algorithm to compute 6D transformations between pairs of frames. Paton and Kosecka [115] applied feature extraction and mapping on RGB-D data with SIFT, motion estimation and outlier rejection with RANSAC, and estimation refinement to compute the position and orientation of a camera. Correspondences established between SIFT features could initialize a generalized Iterative Closest Point (ICP) algorithm.

Salas-Moreno et al. [116] proposed a GPGPUparallel 3D object detection algorithm and a pose refinement based on ICP. Their real-time incremental SLAM was designed to work even in large cluttered environments. Prior to SLAM, they created a database of 3D objects with KinectFusion. The scene was represented by a graph, where each node stored the pose of an object with a correspondent entry in the database. Their object level scene description offered a huge representation compression in comparison with the usual reconstruction of the environment into point clouds.

A robust key-frame selection from RGB-D image streams, combined with pose tracking and global optimization based on the depth camera model, vertex-weighted pose estimation, and edge-weighted global optimization, was described by Tang et al. [118].

Most solutions acquire images with structured light or time-of-flight cameras, but stereo cameras can also provide 3D information. For instance, Albrecht and Heide [117] acquired images with a stereo camera and applied ORB-SLAM2 for poses of the keyframes, creating a 3D reconstruction of the environment with OpenCV’s Semi-Global Block Matching (SGBM) algorithm. Then, they condensed the point cloud into a blueprint-like map of the reconstructed building, based on ground and wall segmentation.

Martin et al. [119] applied Monte Carlo based probabilistic self-localization on a map of colored 3D points, organized in an octree. They demonstrated that their algorithm recovered quickly from cases of unknown initial position or kidnappings (the robot was manually displaced from one place of the environment to another).

Table 16 presents the computing capabilities and obtained accuracy for several SLAM based localization solutions that apply low-level image processing on data that contain both color and depth information. It can be observed that some of the researchers evaluated their algorithms only through visual inspection. Even so, inspection of the obtained 3D reconstruction and especially loop closure can demonstrate the performance in the case of SLAM based solutions. This class is reduced to a 3D to 3D matching problem, much less complex than the 3D to 2D matching problem described in Section 3.2.13 and Section 3.2.14. However, the requirement to have a 3D camera both in the configuration stage and in the online phase greatly reduces the applicability of this kind of solution.

#### 3.2.16. Indoor Localization Solutions with a 3D Model of the Environment, 3D Mobile Cameras, Real Features, and Artificial Intelligence

Another class of indoor localization solutions is the one that uses 3D cameras in the configuration stage, to create a reconstruction of the scene with SLAM or other algorithms, but also in the localization stage, applying high level computer vision techniques for computing the position and orientation of the user.

Guclu et al. [121] proposed an SLAM method applied on RGB-D images using a graph based approach. The keyframe autocorrelogram database estimated motion between frames. Keyframes were indexed based on their image autocorrelograms [150], using a priority search k-means tree. Adaptive thresholding was used to increase the robustness of loop closure detection.

Kuang et al. [120] improved ORB-SLAM. A combination between quasi-physical sampling algorithm (based on BING features [151], obtained by SVM training) with depth information was used to pre-process an image for decreasing the computing time of the ORB algorithm. Then, improved KD-trees were used to increase the matching speed of the ORB algorithm. Furthermore, using RGB-D images, a 3D dense point cloud map system was constructed, instead of a sparse map from ORB-SLAM.

As can be observed in Table 17, the use of 3D cameras can improve the accuracy of known localization methods such as ORB-SLAM or ORB-SLAM2. Still, the dimensionality of the data introduces a high computational cost. Furthermore, the lack of 3D training data could represent a limitation of this class.

#### 3.2.17. Indoor Localization Solutions with a 3D Model of the Environment, 2D Mobile Cameras + Other Sensors, Real Features, and Traditional Image Analysis

A hybrid approach that fuses information from 2D cameras and other sensors can be applied on 3D models of the environment as well.

For instance, Wang et al. [46] used RFID readers for an approximate estimation of the location and calculation of 3D image coordinates with low-level image matching.

Kao and Huy [41] combined information from WiFi access points with the K-nearest neighbor method, inertial sensors (accelerometer and gyroscope), and a CMOS camera. They chose ORB features in their SLAM implementation to navigate Bluetooth connected wheeled robots in indoor environments.

Yun et al. [122] saved the WiFi access point information in a configuration stage and assembled the images acquired with an Xtion PRO LIVE depth camera, building a 3D indoor map of the indoor location. In the localization stage, they reduced the per-frame computation by splitting a video frame region into multiple sub-blocks and processing only a sub-block in a rotating sequence at each frame. They applied SIFT based keypoint detection and optical flow for tracking.

Huang et al. [95] applied an extended Kalman filter to fuse data from LSD-SLAM computed on RGB images, ZigBee localization, and IMU sensors (accelerometer, gyroscope, and magnetometer). Ullah et al. [125] combined data from a monocular visual SLAM and an IMU with an unscented Kalman filter. Gerstweiler [45] also fused IMU information with SLAM, using the HyMoTrack framework [153], a hybrid tracking solution that uses multiple clusters of SLAM maps and image markers, anchored in the 3D model.

Chan et al. [124] computed a laser based SLAM and a RBPF based visual SLAM. Perspective trajectories obtained from the laser SLAM were mapped into images, and the essential matrix between two sets of trajectories was combined with the monocular camera based SLAM.

Even if the fusion between sensor data and visual information introduces a high computational load, several solutions achieve real-time frame rates on commodity computers, as can be observed in Table 18.

### 3.3. Discussion

This section draws conclusions from our analysis of the proposed classes of vision based localization solutions, enabling readers to make better informed choices in terms of indoor positioning technologies to accommodate their specific requirements or particularities. While positioning technologies are numerous and do not limit themselves to image processing, vision based solutions have become popular due to the increasing affordability of cameras and their integration in pervasive devices such as smartphones.

Localization methods that use static cameras can benefit from the camera surveillance infrastructure already available in most modern large office and public buildings. Furthermore, since most robotic platforms have RGB or RGB-D cameras, it makes it easier to port visual positioning solutions on the different platforms, enabling their use in assisted living scenarios. Other applications in the autonomous robots domain can take advantage of 2D/3D cameras already integrated in the robots. Smart glasses with cameras can enable a more seamless user experience for indoor localization applications; however, until they reach a wide market adoption, most applications that localize people (especially in the domains of assistive devices and augmented reality) use smartphones.

Even though 2D cameras have larger applicability due to their ubiquity and the dimensionality of the acquired data, 3D cameras have several advantages. 3D cameras offer a depth map of the environment, either obtained from a disparity map computed with stereo matching algorithms or estimated with time-of-flight and structured light technologies. Stereo cameras require optimal lighting conditions and are affected by lens distortion, similar to 2D cameras. Furthermore, depth cannot be estimated in untextured environments through stereo matching. On the other hand, structured light and time-of-flight cameras work even in unlit environments and can estimate the depth regardless of texture properties. Although these cameras are affected by bright light and reflective surfaces, typical indoor environments contain untextured surfaces (especially uniformly painted walls) and are rarely characterized by bright sunlight. Therefore, we considered that among 3D cameras, structured light, and time-of-flight devices are the most suited for indoor applications.

While cameras have many advantages, they are affected by lighting conditions, occlusion, and position changes of objects from the environment. In order to increase localization accuracy or to decrease the computational load of the computer-vision algorithms, visual data can be combined with data from other sensors. Other popular indoor localization solutions are those based on sensors such as WiFi, beacons, and RFID. WiFi based solutions use the received signal strength and the media access control address of access points to determine the position. WiFi based methods also enjoy the advantage of using existing infrastructure in buildings, as WiFi access points are even more widely available in buildings than camera surveillance systems. While beacon based positioning technologies can reach higher accuracy than WiFi based solutions, they require deploying additional hardware. The RFID technology poses even more limitations in terms of range. Although the positioning algorithms that use sensors such as WiFi, beacons, or RFID have a lower accuracy compared to vision based methods, they also have a lower complexity. Thus, possible localization solutions can benefit from a two-step positioning algorithm: firstly obtaining a quick, approximate location using beacons or WiFi, which tightens the search space of computer vision algorithms; secondly achieving an accurate and also quick location and orientation estimation of the tracked entity.

Vision based indoor localization solutions can detect fiducial markers or features from real images of the environment. The use of artificial markers enables extremely fast detection and position estimation. Due to the geometric properties of the fiducial markers and their accurate localization with 2D cameras, the use of 3D cameras is unjustified. The biggest disadvantage of using markers is the requirement of covering the space with synthetic images, which can have a negative visual impact on the environment. Therefore, the applicability of such solutions is reduced. Features or semantic objects detected from real images of the environment do not visually influence the environment. However, setting up a database of features/images, annotated with position and orientation information, or creating a 3D model of the environment represent cumbersome processes. Furthermore, changes in the environment, such as rearranging furniture or paintings and posters, would require another configuration stage for rebuilding the feature/image database or the 3D model of the scene.

Objects or features from images can be detected using traditional image processing or artificial intelligence methods. Traditional image processing methods perform detection by comparing different features that are extracted from the images, and the recognition success depends on the selected features. On the other hand, the artificial intelligence methods used for object recognition are mainly based on convolutional neural networks, thus not needing to select the features for recognizing objects, as convolutional neural networks learn specific objects directly from images. One disadvantage of these networks is the high number of images required for training the network. Training data can be obtained either by manual acquisition and annotation or from publicly available datasets. Public datasets are very helpful; however, they are only a few available (especially containing 3D data), and they are limited to several semantic classes.

## 4. Benchmarks

The evaluation methods used for the indoor localization solutions presented in this paper differ. Some are based on visual inspection, some on testing the solutions in certain scenarios or testbeds, and some on using public datasets. This section presents benchmarks created for evaluating localization methods. They can differ based on the input information, which consists of monocular or RGB-D images and WiFi, along with other sensor readings. Some testbeds are designed with the purpose of evaluating only the location and orientation accuracy, while others can also evaluate the correctness of 3D reconstructions in SLAM based methods. Several research papers released to the public a series of datasets and evaluation tools [139,155,156], while others proposed reference systems that can be used for testing the accuracy of localization solutions [157], the latter enabling fair comparisons of existing localization systems in similar conditions [158,159,160].

Sturm et al. [139] proposed a benchmark for the evaluation of RGB-D SLAM based localization solutions. The TUM dataset and this benchmark represent a popular testing tool. This is noticeable in the tables in Section 3. The database consists of images acquired with a Kinect sensor, containing both color and depth information, at a resolution of 640×480. They provide ground truth trajectories that are computed with a motion-capture system composed of eight cameras that acquire images at 100 Hz. These sequences cover a variety of cases, from short to long trajectories, with or without loop closure. Their benchmark offers automatic evaluation tools to assess the drift of visual odometry solutions, as well as the global pose error of SLAM based methods. Another popular benchmark, which contains the ICL-NUIM dataset, was provided by Handa et al. [138]. The database consists of RGB-D frames within synthetically generated scenes with the point of view of handheld cameras. It contains ground truth camera poses and surface models, which enable not only the evaluation of localization solutions, but also of the surface reconstruction accuracy of SLAM based methods. Sun et al. [155] proposed a dataset for evaluating computer vision based localization solutions that compute the pose of a 2D camera with respect to a 3D representation of the scene. Their database contained training data acquired with cameras and a LiDAR scanner, which measured the distance to a target by illuminating it with a laser light and computing the difference in return times for the reflected light. The LiDAR point clouds were used as a reference in a semi-automatic localization workflow that estimated the camera pose with six degrees of freedom. They compared this dataset with several image based localization datasets produced with the SfM algorithm [136,161,162], claiming the creation of a point cloud with much higher density and precision. EgoCart [156] is another benchmark dataset, comprising of almost 20,000 RGB-D images, annotated with information of the camera position and orientation. The authors made the dataset public, along with the evaluation (in terms of accuracy, computing time, and memory requirements) of various machine learning based localization solutions [163,164,165,166].

Schmitt et al. [157] presented an indoor localization system that relied on visual information provided by two Microsoft Kinect devices and on wheel-odometry data acquired with a Roomba robot. Within the ROS framework, they enhanced a pre-drawn floor plan with SLAM, achieving an average error of 6.7 cm for the position estimation. The authors claimed that the accuracy was sufficient to use the system as a reference, when testing the performance of other systems. Their robot could carry the components of the system under test and collect data, without interfering with the localization process.

Ibragimov and Afanasyev [158] analyzed the feasibility of using different visual SLAM based localization methods for robot systems in homogeneous indoor spaces. Their evaluation testbed was built with a monocular camera, a LiDAR sensor, a ZEDstereo camera, and a Kinect device. LIDAR based HECTORSLAM and a tape measure are considered ground truth for comparing trajectories obtained with ORB-SLAM [152], Dense Piecewise Parallel Tracking and Mapping (DPPTAM) [167], Stereolabs’ ZedFu [168] 3D mapping tool, and Real-Time Appearance Based Mapping (RTAB-MAP) [169]. Filipenko and Afanasyev [159] compared various SLAM based methods integrated in the ROS framework: GMapping [170], Parallel Tracking and Mapping (PTAM) [171], HectorSLAM [172], Semi-direct Visual Odometry (SVO) [173], LSD-SLAM [174], RTAB-MAP [169], ORB-SLAM [152], DPPTAM [167], Direct Sparse Odometry (DSO) [175], Cartographer [176], and Stereo Parallel Tracking and Mapping (S-PTAM) [177]. They built a robot equipped with a 2D LiDAR, a monocular camera, and a ZED stereo camera. ATE was the chosen means of evaluation, represented through statistical metrics such as RMSE or the standard deviation. Ragot et al. [160] performed an evaluation of two Visual SLAM algorithms, ORB-SLAM2 [148] and RTAB-MAP [169,178,179]. During the comparison of the two solutions, they used a VICON motion capture system as the ground truth. They performed various experiments, with straight-line, straight-line and back, circular paths, or trajectories containing loop closure.

## 5. Conclusions

Indoor localization has an increasingly vast applicability in domains such as AR, navigation systems, assistive devices (especially for the visually impaired), autonomous robots, surveillance, and monitoring. Since surveillance cameras and smartphone cameras represent a commodity currently, researchers have developed a plethora of indoor localization solutions based on visual input.

This paper offers an overview of the computer vision based indoor localization domain, discussing applications areas, commercial solutions, and benchmarks and presenting some of the most relevant contributions in the area. It also provides a survey of selected positioning solutions, proposing a new classification that organizes the solutions according to the use of known environment data, the sensing devices, the type of detected elements (artificial markers or real features), and the employed localization methods. The research papers selected in the 17 classes of the proposed taxonomy were chosen from prestigious research databases based on their relevance to the domain and publication date, and their purpose was to be illustrative for the reader in terms of the indoor positioning technologies. Since many relevant papers were too recent to have a considerable number of citations, we decided to not use this criterion for selection. The focus was on providing short descriptions of the solutions, highlighting the advantages and disadvantages and presenting the achieved performances (in terms of running time and location estimation accuracy), along with the properties of the datasets used for testing.

Table 2, Table 3, Table 4, Table 5, Table 6, Table 7, Table 8, Table 9, Table 10, Table 11, Table 12, Table 13, Table 14, Table 15, Table 16, Table 17 and Table 18 show that many papers did not report computing times and the specifics of their testbeds, and few papers discussed the financial implications of implementing such solutions in real life. The evaluation methodologies for the presented solutions differed. While some used visual observations to test their solutions, others chose to use private datasets. Although public datasets and benchmarks exist, they come with limitations in terms of required environment data and input from cameras, limiting their use to only certain localization solutions.

We considered this paper a good guide to the field of computer vision based indoor localization. Our proposed classification and the selection of localization solutions for each category aimed to allow the reader to easily grasp the advantages and applicability of each class of solutions.

## Figures and Tables

**Table 1:** Classification of computer vision based localization research papers considering the environment data, the sensing devices, the detected elements, and the localization algorithm.

Classification				Research Papers
Environment Data	Sensing Devices	Detected Elements	Localization Method	
Marker/camera position	2D static cameras	Artificial	Image analysis	[31,32]
Marker/camera position	2D static cameras	Real	Image analysis	[60,67,68,69]
Marker/camera position	2D static cameras	Real	AI	[70,71,72,73,74]
Marker/camera position	2D mobile cameras	Artificial	Image analysis	[38,75,76,77,78,79,80,81]
Marker/camera position	3D mobile cameras	Artificial	Image analysis	[82,83]
Marker/camera position	2D cameras, sensors	Artificial	Image analysis	[36,37,84]
Image/feature database	2D mobile cameras	Real	Image analysis	[85,86,87,88]
Image/feature database	2D mobile cameras	Real	AI	[89,90,91]
Image/feature database	3D mobile cameras	Real	AI	[63,92]
Image/feature database	2D cameras, sensors	Real	Image analysis	[93,94,95,96]
Image/feature database	2D cameras, sensors	Real	AI	[97,98,99,100,101]
Image/feature database	3D cameras, sensors	Real	Image analysis	[102,103]
3D model	2D mobile cameras	Real	Image analysis	[29,104,105,106,107,108,109,110,111]
3D model	2D mobile cameras	Real	AI	[112,113]
3D model	3D mobile cameras	Real	Image analysis	[114,115,116,117,118,119]
3D model	3D mobile cameras	Real	AI	[120,121]
3D model	2D cameras, sensors	Real	Image analysis	[41,45,46,122,123,124,125]

**Table 2:** Characteristics of indoor localization solutions with 2D static cameras (with known positions), markers, and traditional image analysis.

Research Paper	Dataset Characteristics	Computing Time and Platform	Accuracy
[31]	own dataset: 1 static camera, covering 1.26 m × 1.67 m	avg. 0.2 s per frame on a server	err. between 0.0002 and 0.01 m (max. err.: 1 cm)
[32]	own dataset: 3 rooms, each with 1 IP camera	real-time	observational

**Table 3:** Characteristics of indoor localization solutions with 2D static cameras (with known positions), real features, and traditional image analysis.

Research Paper	Dataset Characteristics	Computing Time and Platform	Accuracy
[67]	public datasets: PETS2009 [126], TUD-Stadtmitte [127]	approximately 140 ms on Intel Core2Quad 2.66 GHz with 8 GB RAM	Multiple Object Tracking Accuracy (MOTA): 87.8% on the PETS2009 and 64.2% on the TUD-Stadtmitte
[69]	own dataset: indoor space with 2.2 m × 6 m, images with 320×240 pixels from 2 cameras	-	less than 7.1 cm
[68]	own dataset: 12,690 frames acquired with 3 cameras; public dataset: PETS2001	-	95.7% hit rate and 96.5% precision
[60]	own dataset: office with 5.1 m × 8.5 m × 2.7 m	-	mean err. of 0.37 m

**Table 4:** Characteristics of indoor localization solutions with 2D static cameras (with known positions), real features, and artificial intelligence.

Research Paper	Dataset Characteristics	Computing Time and Platform	Accuracy
[70]	public datasets: PETS 2009 (City Center), EPFLterrace dataset	-	Ground Position Error (GPE) metric with total err. rate 0.122/0.131, Projected Position Error (PPE) metric with total err. rate 0.107/0.140
[71]	training: 1542 images (own) + 25,608 images from MS COCO; evaluation: 1400 images/robot type + 110/pattern	50 Hz on a GPU and 10 Hz on a CPU	detection rate between 70% and 97.9%; orientation err. between 1.6 and 11.9 degrees
[72]	own dataset: 47 employees, 18 rooms and 6 cubicles, 960 ceiling images	2.8 s per image (offline computation)	88.2% accuracy for identifying locations
[73]	own dataset: over 2100 frames in 42 scenarios	6.25 fps on Jetson TX2	approximately 45 cm mean err.
[74]	own dataset: office room with 1 camera and supermarket with 6 cameras	5 fps on a server	detection success rate of 90% and avg. localization err. of 14.32 cm

**Table 5:** Characteristics of indoor localization solutions with 2D mobile cameras, markers with known positions, and traditional image analysis.

Research Paper	Dataset Characteristics	Computing Time and Platform	Accuracy
[75]	own dataset: classroom with area 2.4 m × 1.8 m and 4 QR codes	Nexus 4 Google (fps not mentioned)	localization err. 6–8 cm, heading direction err. 1.2 angles
[76]	own dataset, simplified and complex scenarios	47 ms for QR code extraction on a Raspberry Pi 2	complex scenario: planar position err. 17.5 cm, 3D pose estimation self-localization err. 10.4 cm
[77]	public dataset proposed by Mikolajczyk and Schmid [134], 4 image pairs	0.11 s, 0.16 s, 0.27 s, 0.14 s; 1–2 iterations to reach the threshold similarity	threshold similarity 0.8
[38]	own dataset: hall with 6 QR codes and 2 possible trajectories (circular and 8-shape)	10 Hz on Linux Ubuntu 12.04 OS running ROS framework	err. for circular path 0.2 m, err. for 8-shape path 0.14 m, orientation err. 0.267 radians
[78]	own dataset acquired with an RGB camera fixed on an FLIR Pan Tilt Unit mounted on a mobile platform with an SICK s300 laser scanner (ground truth), markers placed on a wall	0.07 s avg processing time of a scene with 550 markers (up to 200 times faster than AprilTags)	avg. error of angle estimates: 0.02 rad.for pitch/roll
[79]	own dataset: space covered with 4 × 4 QR codes, placed 50 cm apart	-	the robot can travel more than 7 times on the same route
[80]	own dataset, images of resolution 1280 × 720	18.1 ms on an image with a cluttered scene and a single marker, using a single core 3.70 GHz Intel Xeon	less than 0.6 degrees std. dev. for rotation, less than 0.4 cm std. dev. for translation
[81]	own dataset in an academic building, four different paths, markers on the ceiling, guidance test with 10 blindfolded users	-	0 miss detections, 2 false detections out of 40 tests

**Table 6:** Characteristics of indoor localization solutions with 3D mobile cameras, markers with known positions, and traditional image analysis.

Research Paper	Dataset Characteristics	Computing Time and Platform	Accuracy
[82]	own dataset	-	distance from the camera to the QR code: 1 cm err.
[83]	own dataset	real time	maximum distance and angles from which the robot can see the QR code are: 270 cm and 51∘.

**Table 7:** Characteristics of indoor localization solutions with 2D cameras + other sensors, markers with known positions, and traditional image analysis.

Research Paper	Dataset Characteristics	Computing Time and Platform	Accuracy
[37]	own dataset	less than 4 ms per frame; convergence time 18 s	less than 0.2 m for position and less than 0.1 orientation for EHF
[36]	own dataset	computational load increases if dead reckoning is invoked with IMU sensors	visual (performance affected if dead reckoning is not used)
[84]	own dataset: corridor with 100 m × 2.25 m and hall with 14 m × 6.5 m	-	accuracy is within 2 m 80% of the time

**Table 8:** Characteristics of indoor localization solutions with real image/feature databases, 2D mobile cameras, and traditional image analysis.

Research Paper	Dataset Characteristics	Computing Time and Platform	Accuracy
[85]	own datasets: 862 frames, 3674 frames	single CPU/Kepler K 20 chip: 20 ms/1.13 ms for a database of 1000 frames, 160 ms/8.82 ms for 8000 frames	within 2 m in most cases
[86]	own datasets: hallways 15 m long	-	estimated moving speed compared to ground truth: max absolute err. 0.0643 m/s, RMSE for speed 0.24–0.37 m/s, RMSE for distance 0.16–0.23 m
[87]	own dataset: 1866 images, 40 key frames	-	-
[88]	own dataset: over 90,000 annotated frames out of 60 videos from six corridors (approximately 3.5 km of data)	-	4 m avg. absolute err. for HOG3D and 1.3 m for SF GABOR, over a 50 m traveling distance

**Table 9:** Characteristics of indoor localization solutions with real image/feature databases, 2D mobile cameras, and artificial intelligence.

Research Paper	Dataset Characteristics	Computing Time and Platform	Accuracy
[89]	own dataset: 1800 images from 30 different locations, 480 indoor videos of buildings (each lasting around 2–3 s), public dataset: Dubrovnik [136]	0.00092 s for image based localization and 0.0012 s for the video based method	95.56%/94.44% accuracy for location/orientation with image based localization and 98% with the video based method
[90]	own dataset: 302 training images, resolution 3024 × 4032	object detection phase takes 0.3 s	location accuracy is within 1 m
[91]	own dataset: 112,919 compound images (composed of 4 images taken by 4 Google Nexus phones) of resolution 224 × 224	close to real-time	avg. median err. after a 20 step moving for compound images is 12.1 cm

**Table 10:** Characteristics of indoor localization solutions with real image/feature databases, 3D mobile cameras, and artificial intelligence.

Research Paper	Dataset Characteristics	Computing Time and Platform	Accuracy
[92]	ICL-NUIMdataset [138] and TUM dataset [139]	296 ms to find the most similar frame and 277 ms to estimate the final pose on Intel Xeon E5-1650 v3 CPU 3.5 GHz, NVidia TITAN GPU	more than 80% of the images are localized within 2.5 degrees and more than 90% are localized within 0.3 m
[63]	ICL-NUIM dataset [138]	-	0.51 m living room, 0.41 m office

**Table 11:** Characteristics of indoor localization solutions with real image/feature databases, 2D cameras + other sensors, and traditional image analysis.

Research Paper	Dataset Characteristics	Computing Time and Platform	Accuracy
[93]	own dataset: 75 location images from 1.5–2 m distance	query time 40–230 ms	mean distance err. rate is 2.5/2.21 m for extended distance estimation method/hybrid approach
[94]	own dataset	90 ms FAST-SURF, 100–130 ms indoor positioning, 3–7 ms character detection, 30–45 ms tracking and registration on Honor 3C smartphone	-
[95]	own dataset (offices and hallways)	0.5 s per frame	90% of location and orientation errors are within 25 cm and 2 degrees
[96]	-	iPhone5s, iPhone X, LG Nexus 5X, Samsung Galaxy S7, S9, Huawei Mate tablet	best results, on Samsung S9: 4.5 deg. avg. rotation and 250 mm position err. from 1 m in front of the marker

**Table 12:** Characteristics of indoor localization solutions with real image/feature databases, 2D cameras + other sensors, and artificial intelligence.

Research Paper	Dataset Characteristics	Computing Time and Platform	Accuracy
[97]	own dataset, 3 blind volunteers	2 fps on a laptop	visual inspection
[98]	own dataset, 5 people with different weight and height, walking at 3 different speeds, on two tracks (straight or zig-zag)	real time	93% accuracy in case of normal speed
[99]	own dataset: office floor 51 m × 20 m × 2.7 m and 7 WiFi routers	-	panoramic camera based method: mean err. for localization 0.84 m, cumulative probability within localization err. of 1 m/2 m is 70%/86%
[100]	own dataset: room-level environment and open large environment	4 s per image (0.8 s fingerprint location on server, 2.9 s image location on smartphone, 1 s data transmission)	less than 0.6 m avg. location err. and less than 6 degrees avg. direction err., 90% location deviations are less than 1 m
[101]	own dataset: 50 m${}^{2}$ office and 2 cases (with and without line-of-sight); ground divided into 42 reference points	real time on Intel5300 NIC laptop with 3 antennas as signal receiver and Ubuntu server with Intel Xeon e5-2609 CPU, GeForce GTX TITAN X GPU and 256 GB RAM	avg. position err. is 0.98/1.46 m for line-of-sight/none line-of-sight

**Table 13:** Characteristics of indoor localization solutions with real image/feature databases, 3D mobile cameras + other sensors, and traditional image analysis.

Research Paper	Dataset Characteristics	Computing Time and Platform	Accuracy
[102]	own dataset: small number of experiments and short tested trajectories	real time	avg. err. in the X-axis direction is 0.06 m with IMU
[103]	own dataset	real time	translation error: 0.1043 m, rotation err.: 6.6571 degrees for static environments; translation err.: 0.0431 m ± 0.0080 m, rotation err.: 2.3239 degrees ± 0.4241 degrees for dynamic environments

**Table 14:** Characteristics of indoor localization solutions with an existing/generated 3D model of the environment, 2D mobile cameras, real features, and traditional image analysis.

Research Paper	Dataset Characteristics	Computing Time and Platform	Accuracy
[29]	own dataset: simple experiment with obstacles along a route	real time on a single CPU	visual inspection
[104]	own dataset: images of resolution 320×240	3 fps for SURF on 2.20 GHz dual-core computer	90% detection success rate and 14.32 cm avg. localization err.
[105]	3 own datasets: images of resolution 640×480 (from Galaxy S4 camera); public dataset: feature set of Liang et al. [144]	avg. search time for Dataset 1 (176 frames) is 10 ms and for Dataset 4 (285 frames) is 28 ms	80–100% accuracy, depending on dataset; 0.173–0.232 m localization error
[106]	own dataset: reconstruction with RGBD-SLAM, Dataset 1 (50 frames, 139 mp), dataset 2 (33 frames, 37 mp)	avg. localization time is 0.72 s per frame on an Intel Core i7 with 8 GB RAM	avg. localization error is less than 10 mm: translation err. for Dataset 1 is 0.9–35 mm and for Dataset 2 is 0.3–17 mm
[107]	own dataset: office environment, with 154 m long route	images captured at 0.8 Hz using a smartphone camera, computing time not mentioned	1.5 degrees mean accuracy for visual gyroscope, 0.3 m/s mean accuracy for visual odometer, 1.8 m localization err.
[108]	own dataset: synthetic scenes (20 m × 20 m scene with 88 lines and 160 points, 794 generated images); public dataset: Biccoca_2009 [145]	0.5–1 s for MATLAB version, 25.8 ms avg. running time for C++ version	0.79 accuracy err. in position on a 967 m path; 0.2 m accuracy err. for synthetic scenes
[109]	own dataset: Guangdong Key Laboratory, Shantou University	-	only visual inspection, in comparison with the RGB-D SLAM method [146]
[110]	own dataset: indoor environment; public dataset: Karlsruhe outdoor datasets [147]	real time on an i7 processor	94–98% distance and depth measurements accuracy; absolute err. of 5.72/9.63 m for 2 outdoor datasets and 4.07/1.35 cm for 2 indoor datasets
[111]	own datasets: office building (400 m${}^{2}$), gymnasium (1000 m${}^{2}$), and a shopping mall (6000 m${}^{2}$), 21 navigation paths, 274 checkpoints	approximately 0.1 s for relocalization and navigation on Huawei P10, Nexus 6, Nexus 7, Lenovo Phab2 pro	98.6% immediate NSR, 93.1% NSR after 1 week, 83.4% NSR after 2 weeks

**Table 15:** Characteristics of indoor localization solutions with an existing/generated 3D model of the environment, 2D mobile cameras, real features, and artificial intelligence.

Research Paper	Dataset Characteristics	Computing Time and Platform	Accuracy
[112]	public dataset: TUM Dynamic Object dataset (RGB images, depth information, ground truth trajectory)	5fps for Mask-RCNN on NVidia Tesla M40 GPU [149]	RMSE between 0.006134 and 0.036156
[113]	own dataset: 370 m from route; public datasets: TUM dynamic dataset, KITTI dataset (outdoor large scenarios)	real time	the trajectory RMSE err. is 2.29 m, the accuracy is 7.48–62.33% higher than ORB-SLAM2 [148]

**Table 16:** Characteristics of indoor localization solutions with an existing/generated 3D model of the environment, 3D mobile cameras, real features, and traditional image analysis.

Research Paper	Dataset Characteristics	Computing Time and Platform	Accuracy
[114]	own dataset: a room, check if the virtual objects have the same size as the real ones	3–4 fps for map building on a laptop with i7-720qm CPU	difference in dimensions between 3D reconstructed and real objects: dm/cm level accuracy
[115]	own dataset: barren office hallway; public dataset: TUM dataset [139]	-	0.1–1.5 m translation RPE, 2–18 degrees rotational RPE, 0.02–1.1 m ATE
[116]	own dataset: room of size 15 × 10 × 3 m${}^{3}$	20 fps on a gaming laptop	visual inspection, checking loop closure
[117]	own dataset: a path of 70 m through a building	25 Hz	visual inspection
[118]	own datasets: taken with a handheld structure sensor; public datasets: Freiburg Benchmark, TUM dataset [139]	5 fps	0.011–0.062 RMSE of ATE for public datasets; 1.4–4.1 cm closing distance and 1.08–3.32 degrees closure angle for own datasets
[119]	own dataset collected with 2 wheeled robots (RB-1 and Kobuki) with Asus Xtion RGB-D sensors	less than 30 s	tracking mode (robot starts from a known position): x-mean: 0.082 m, y-mean 0.078 m; global mode (robot starts from unknown position): x-mean 0.27 m, y-mean 0.43 m

**Table 17:** Characteristics of indoor localization solutions with an existing/generated 3D model of the environment, 3D mobile cameras, real features, and artificial intelligence.

Research Paper	Dataset Characteristics	Computing Time and Platform	Accuracy
[120]	public dataset: TUM dataset [139]	37.753 ms per frame on Intel i5 2.0 GHz CPU with 3 GB RAM	0.015 and 0.103 RMSE for the error size of the posture, better than ORB-SLAM [152]
[121]	public dataset: ICL-NUIM [138] and TUM dataset [139]	119.0 ms (average) on a desktop PC running Ubuntu 12.04 with an Intel Core i7-2600 CPU at 3.40 GHz and 8 GBRAM	Absolute Trajectory Error ATE: 1 cm–5 cm, mostly competing with ORB-SLAM2 [152]

**Table 18:** Characteristics of indoor localization solutions with an existing/generated 3D model of the environment, 2D mobile cameras + other sensors, real features, and traditional image analysis.

Research Paper	Dataset Characteristics	Computing Time and Platform	Accuracy
[46]	own dataset	-	visual evaluation
[41]	own dataset: robot moves along a specific path in a lab room, QVGA resolution	200 ms for basic image processing on LG P970, whole pipeline processed offline (manual extraction of features)	position err. converges from 35–50 cm to less than 3 m
[122]	own dataset: 120 m indoor hallway with 5200 video frames of size 640×480	from 3.2 fps to 23.3 fps on commodity laptop (2.6 GHz quad-core CPU and 4 GB RAM)	0.17 m position err.
[95]	own dataset	-	visual inspection
[124]	own dataset	map building and fusion process: real time on Intel Core i7-8550U CPU	relative error of ORB-SLAM2 [148] calibrated with proposed mapping matrix is less than 5%
[125]	own dataset and public dataset: EuRoCdataset [154]	real time on an embedded board (1.92 GHz processor and 2 GB DDR3L RAM)	0.01–0.15 m position err. for own dataset; 0.234 m max. err. for EuRoC dataset
[45]	own dataset: Vienna airport, path of 200 m	23 s for the proposed method to complete a guiding task	visual inspection
